# Supplementary material for: Netcombin: An algorithm for constructing optimal phylogenetic network from rooted triplets
Source: PLoS One. 2020 Sep 18;15(9):e0227842. doi: 10.1371/journal.pone.0227842 (PMC7500971; doi:10.1371/journal.pone.0227842)
Supplement: S4 File — (ZIP) [file pone.0227842.s004.zip › SequenceData-Triplet/sequenceData-10-20-30-0-Triplet/length 400, numseq 30, rate 10^-7/4 seq.pdf]

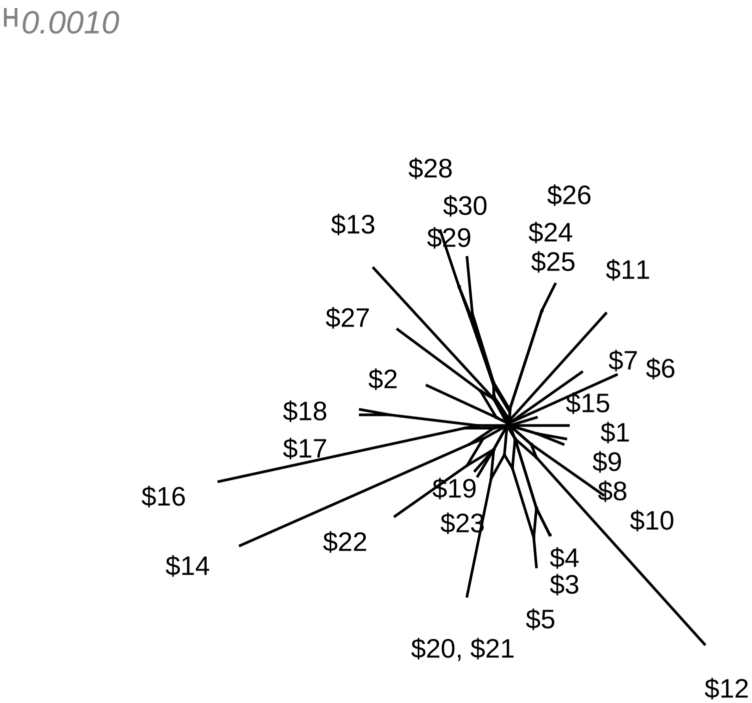

0.0010
$28
$30
$26
$13
$29
$24
$25
$11
$27
$7
$6
$2
$15
$18
$1
$17
$9
$8
$19
$16
$10
$23
$22
$4
$14
$3
$5
$20, $21
$12
